# Crystal structure and Hirshfeld surface analysis of saflufenacil

**DOI:** 10.1107/S2056989026004585

**Published:** 2026-05-12

**Authors:** Jiyong Liu

**Affiliations:** a Department of Chemistry Zhejiang University,Hangzhou 310058 People’s Republic of China; Universidad de Los Andes Mérida, Venezuela

**Keywords:** Saflufenacil, crystal structure, Hirshfeld surface, energy framework, hydrogen bonding

## Abstract

The crystal structure of saflufenacil or 2-chloro-4-fluoro-*N*-(*N*-isopropyl-*N*-methyl­sulfamo­yl)-5-[3-methyl-2,6-dioxo-4-(tri­fluoro­meth­yl)-1,2,3,6-tetra­hydro­pyrimidin-1-yl]benzamide, C_17_H_17_ClF_4_N_4_O_5_S, has been determined by single-crystal X-ray diffraction. The compound crystallizes in the monoclinic space group *P*2_1_/*c.* The asymmetric unit contains one mol­ecule of Saflufenacil. The tri­fluoro­methyl-substituted di­hydro­pyrimidine ring is essentially planar, while the dihedral angle between this ring and the central benzene ring is 86.70 (6)°, indicating an almost perpendicular orientation. In the crystal, N—H⋯O hydrogen bonds link adjacent mol­ecules into infinite chains extending along the *b-*axis direction, which are further assembled into two-dimensional layers *via* weak C—H⋯O inter­actions. Hirshfeld surface analysis reveals that H⋯O/O⋯H, H⋯H, H⋯F/F⋯H and H⋯C/C⋯H contacts contribute 26.6%, 20.8%, 15.4% and 8.7%, respectively, to the total surface area. Energy framework calculations confirm a two-dimensional inter­action network with a high total energy, consistent with the close-packing arrangement in the crystal.

## Abstract 2

The crystal structure of saflufenacil is reported for the first time, revealing an almost perpendicular arrangement of the pyrimidine and benzene rings and a two-dimensional hydrogen-bonded network that is qu­anti­tatively characterized by Hirshfeld surface and energy framework analyses.

## 1. Chemical context

Saflufenacil (development code BAS800H, marketed under trade names including Kixor) is a pyrimidine­dione herbicide discovered and developed by BASF. It was launched commercially in 2009 and is registered for use on more than 30 crops, including corn, soybean, sorghum, wheat, cotton, and fruit trees, for the control of nearly 100 broad-leaf weed species. Saflufenacil exhibits outstanding efficacy against weed populations that have developed resistance to widely used herbicides such as triazines, glyphosate, and acetolactate synthase (ALS) inhibitors (Grossmann *et al.*, 2010 ▸). Its mol­ecular structure can be divided into three distinct moieties: a pyrimidine­dione ring, a central benzene ring, and a sulfonamide side chain.
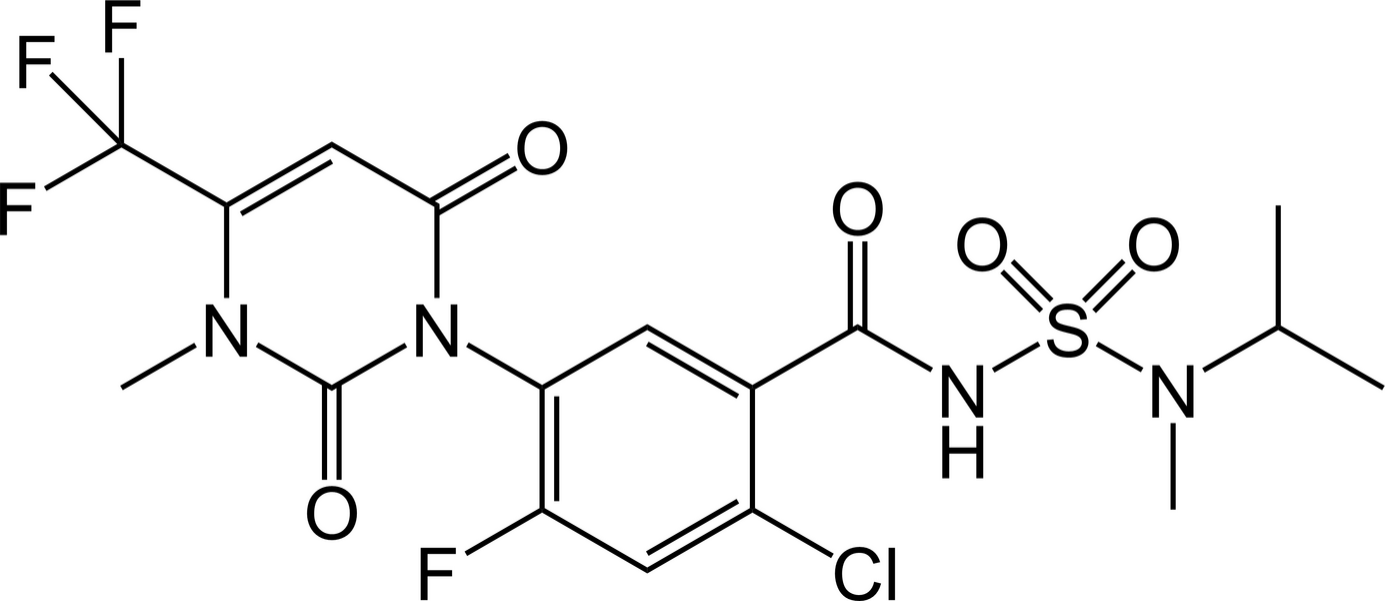


Although several crystalline forms of saflufenacil have been described in the patent literature (Schmidt *et al.*, 2007*a* ▸,*b* ▸), including a crystalline anhydrate, two crystalline hydrate forms, and an aceto­nitrile solvate, no detailed investigation of its crystal structure has been reported to date. In this context, we present herein the crystal structure of saflufenacil along with a comprehensive Hirshfeld surface analysis to elucidate its mol­ecular packing and inter­molecular inter­action network.

## 2. Structural commentary

**Figure 1:**
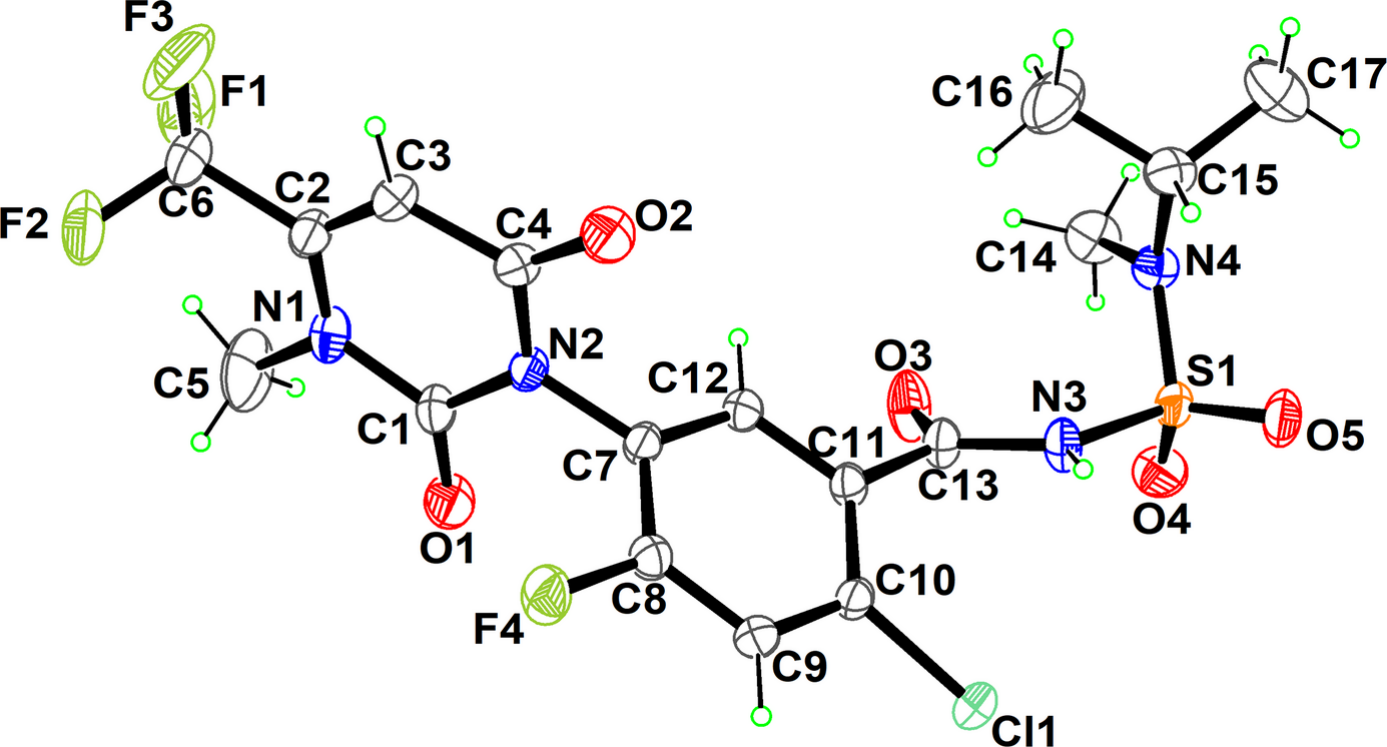
The mol­ecular structure of the title compound showing the atom-labeling scheme. Displacement ellipsoids are drawn at the 30% probability level.

The title compound crystallizes in the monoclinic *P*2_1_/c space group with one mol­ecule in the asymmetric unit (Fig. 1 ▸). The compound consists of a tri­fluoro­methyl-substituted 3-methyl-2,6-dioxo-3,6-di­hydro­pyrimidine ring (*A*, O1/O2/N1/N2/C1–C4), a fluoro- and chloro-substituted benzamide moiety (*B*, F4/Cl1/C7–C12), and an alkyl-substituted sulfonamide group. The di­hydro­pyrimidine ring is essentially planar, with an r.m.s. deviation of 0.027 Å. Atoms O1, O2, C5 and C6 deviate from the best least-squares plane through ring *A* by −0.153 (3), 0.054 (3), 0.123 (5) and 0.030 (4) Å, respectively. Rings *A* and *B* are nearly perpendicular to each other, subtending a dihedral angle of 86.70 (6)°. The torsion angle C13—N3—S1—N4 is 63.4 (2)°. No unusual bond lengths or bond angles are observed.

## 3. Supra­molecular features

**Figure 2:**
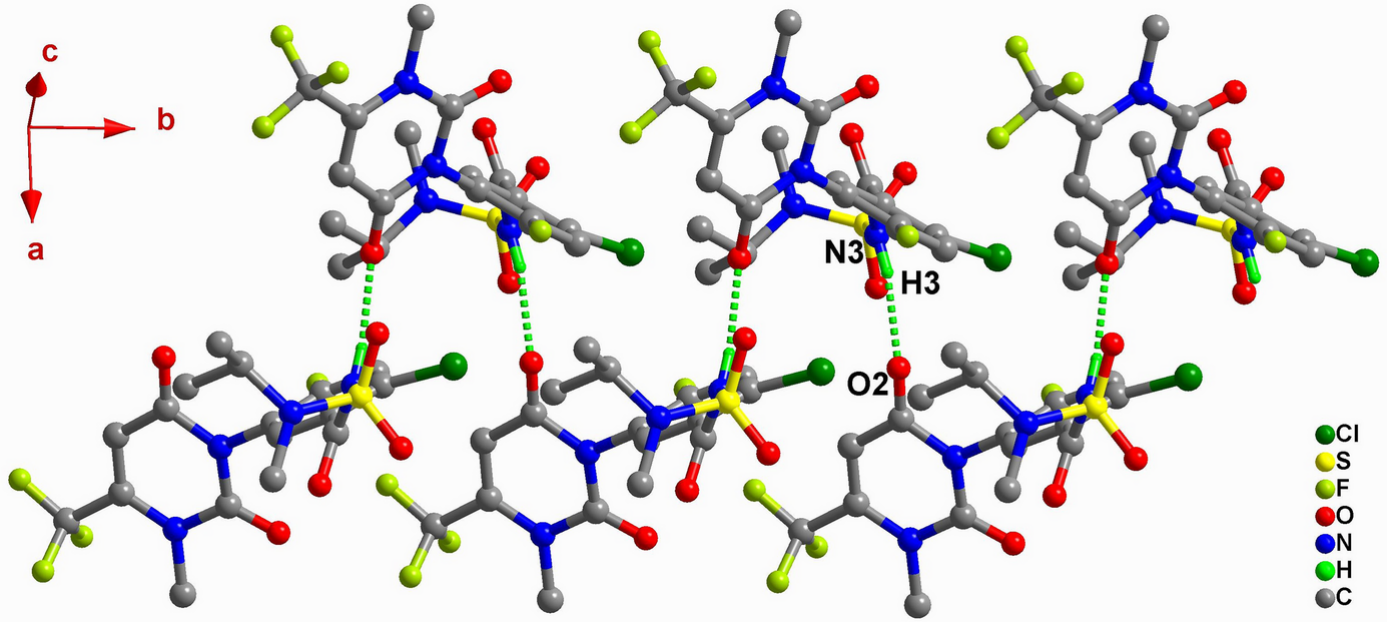
The mol­ecular chain along the *b-*axis direction. Hydrogen bonds are shown as dashed lines.

**Figure 3:**
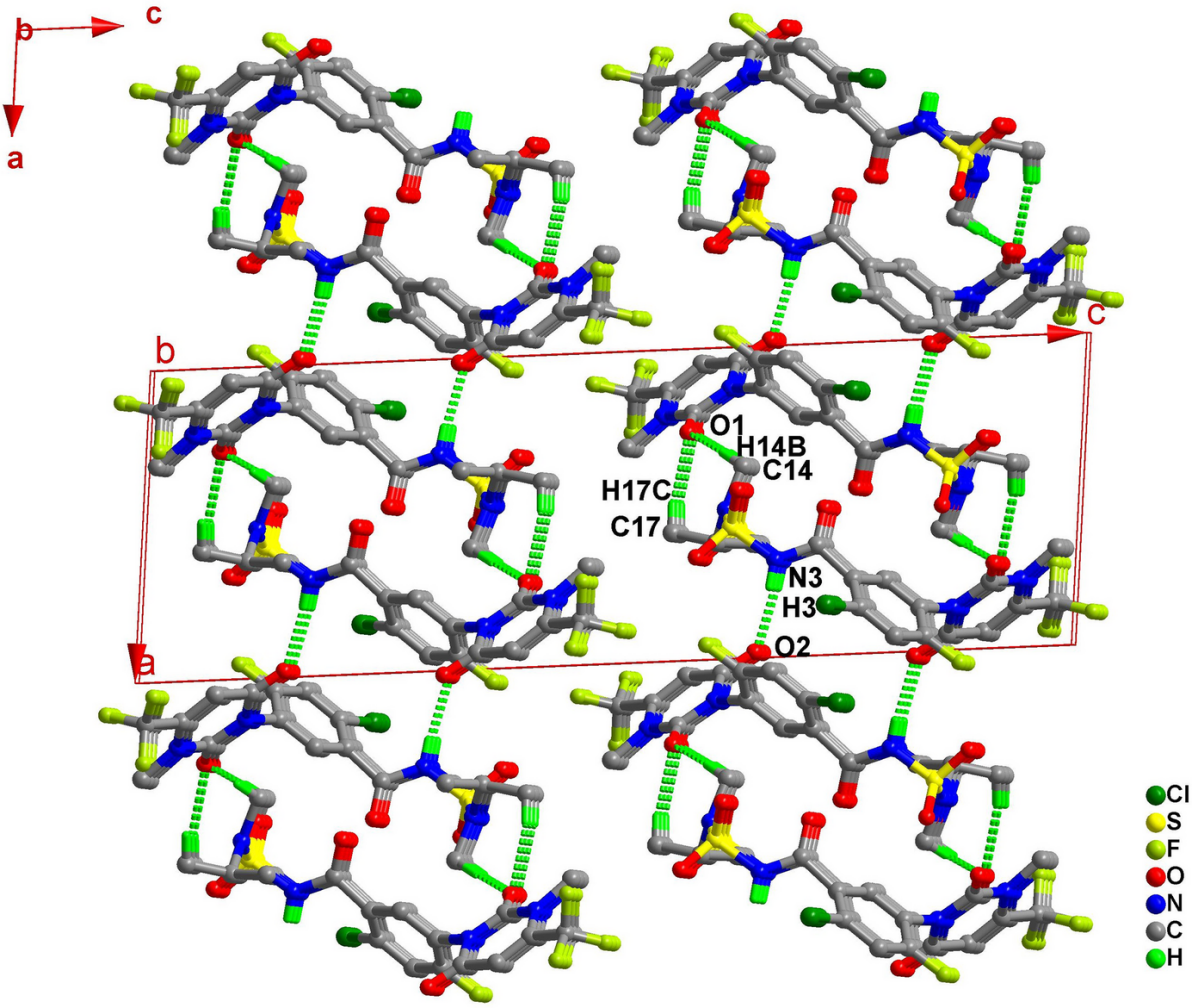
The mol­ecular packing of Saflufenacil viewed down the *b* axis.

In the crystal, the benzamide nitro­gen atom N3 acts as a hydrogen-bond donor, and the carbonyl oxygen atom O2 of the dioxodi­hydro­pyrimidine ring acts as an acceptor. The resulting hydrogen bond (N3—H3⋯O2^ii^) links saflufenacil mol­ecules into an infinite hydrogen-bonded chain extending along the *b*-axis direction (Table 1 ▸, Fig. 2 ▸). Subsequently, through weak C—H⋯O hydrogen bonds (C14—H14*B*⋯O1^i^, C17—H17*C*⋯O1^i^), these chains stack along the *a*-axis direction, forming a two-dimensional hydrogen-bonded layer (Fig. 3 ▸).

**Figure 4:**
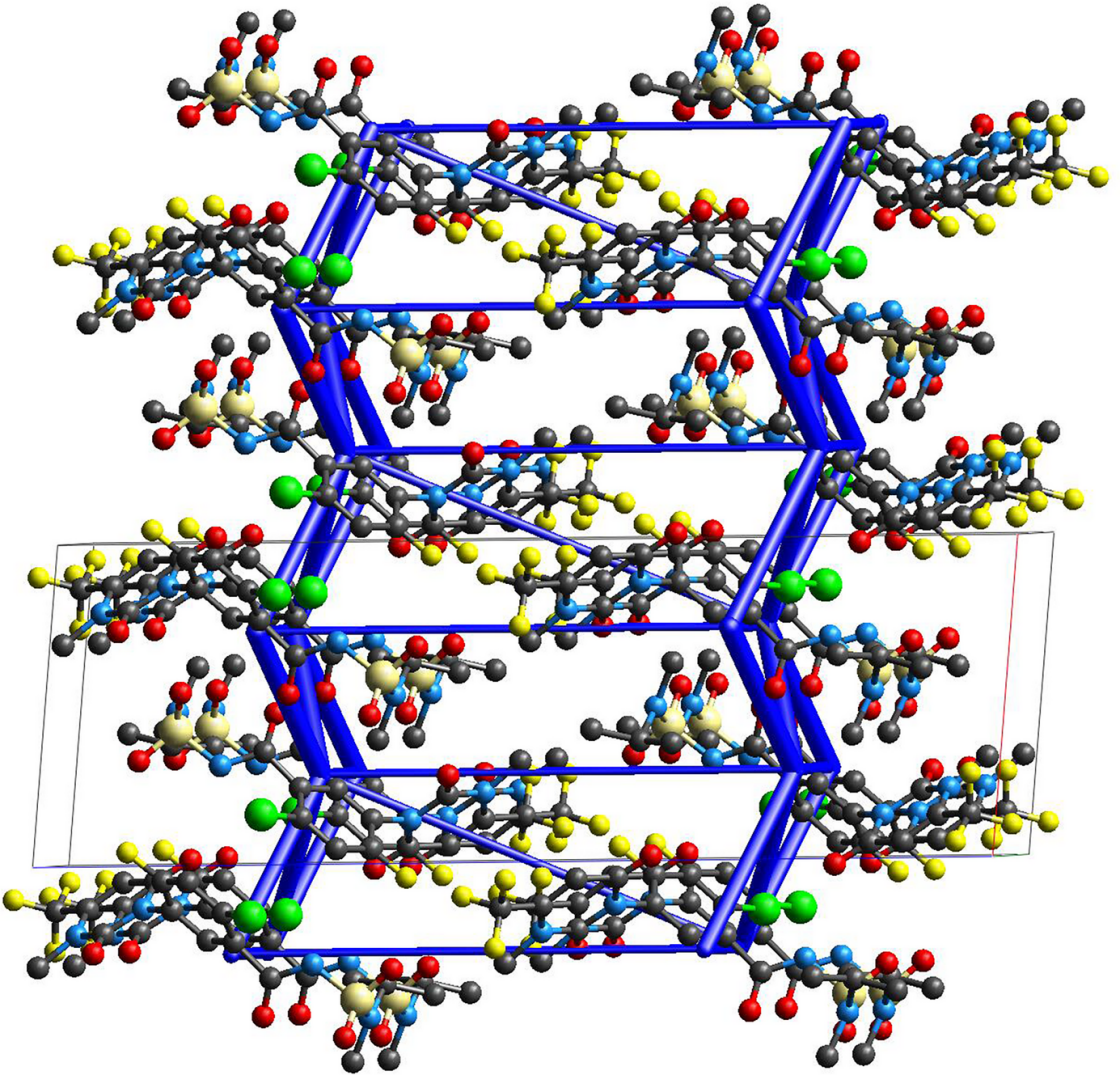
Total energy framework of the title compound, shown along the *b* axis. The cylinder thickness is set to 50 arbitrary units, and inter­action energies below 15 kJ mol^−1^ have been omitted.

To better understand the crystal formation and properties, the energy frameworks of saflufenacil were calculated using the B3LYP/6-31G(d,p) model in *CrystalExplorer21.5* (Spackman *et al.*, 2021 ▸), accompanied by visualization graphics. In these frameworks, the visual cylinders are proportional to the magnitudes of the inter­molecular inter­actions. The first strongest pairwise inter­action is approximately −64.0 kJ mol^−1^, with an inter­molecular distance of about 6.72 Å, mainly contributed by two sets of inter­molecular C—H⋯O hydrogen bonds. The second strongest pairwise inter­action is −51.3 kJ mol^−1^, with an inter­molecular distance of about 6.72 Å, corresponding to the major hydrogen bond N3—H3⋯O2 that forms the hydrogen-bonded chain. The topology of the energy frameworks resemble the crystal packing and form a two-dimensional structure akin to the hydrogen-bond network (Fig. 4 ▸). This structure is characterized by a high total energy value, indicative of the effective packing within the crystal.

## 4. Hirshfeld surface analysis

**Figure 5:**
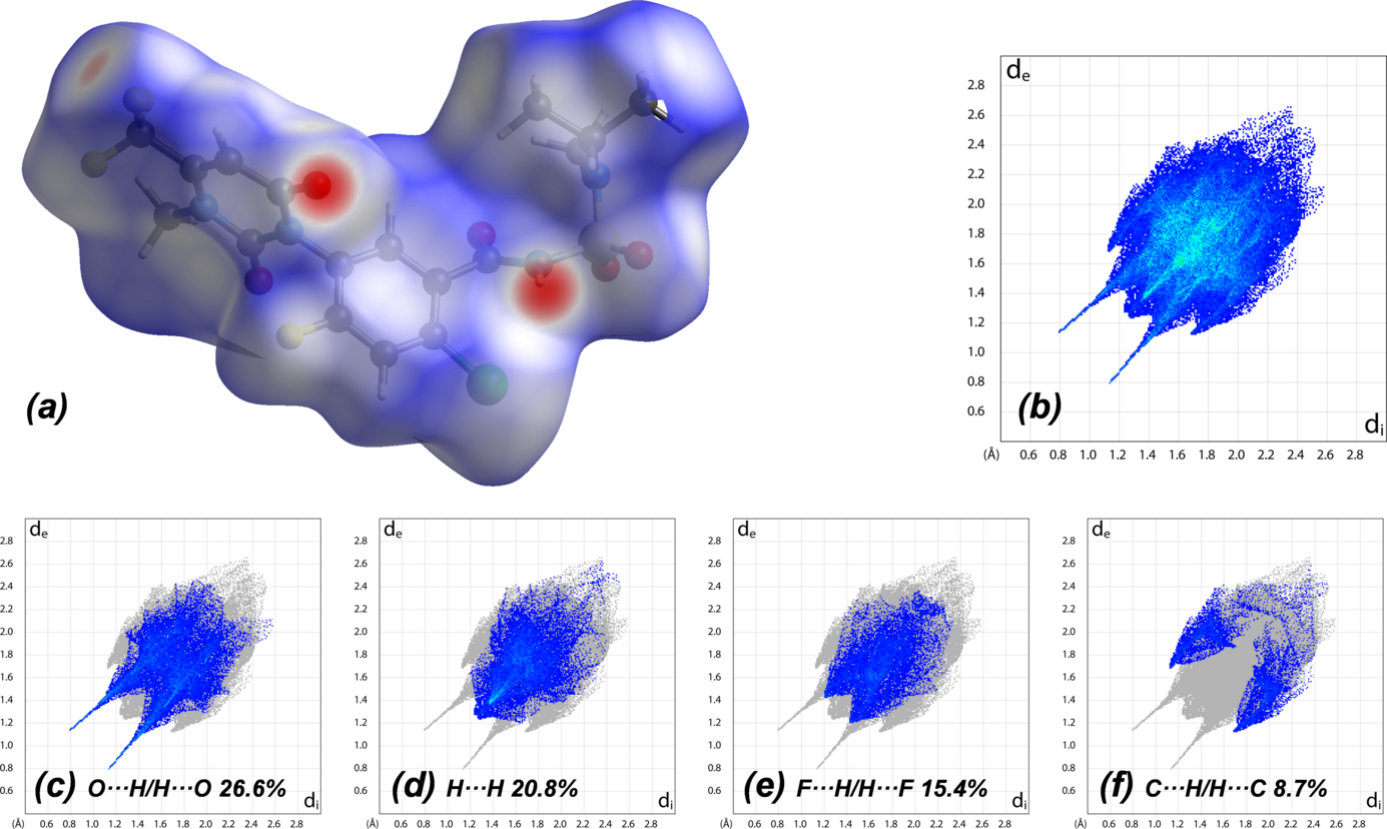
(*a*) View of the three-dimensional Hirshfeld surface of the title compound plotted over *d*_norm_. The two-dimensional fingerprint plots, showing (*b*) all inter­actions, and those delineated into (*c*) O⋯H/H⋯O, (*d*) H⋯H, (*e*) F⋯H/H⋯F and (*f*) C⋯H/H⋯C inter­actions. The *d*_i_ and *d*_e_ values are the closest inter­nal and external distances (in Å) from given points on the Hirshfeld surface.

A Hirshfeld surface (HS) analysis was carried out using *CrystalExplorer21.5* (Spackman *et al.*, 2021 ▸) to visualize the inter­mol­ecular inter­actions in the crystal. Fig. 5 ▸ shows the contact points where the bright-red spots correspond to the respective donors and/or acceptors (Spackman *et al.*, 2002 ▸, 2009 ▸). The white surfaces and the red and blue areas indicate contacts with distances equal, shorter and longer, respectively, than the van der Waals radii. According to the two-dimensional fingerprint plots (McKinnon *et al.*, 2007 ▸), the O⋯H/H⋯O, H⋯H, F⋯H/H⋯F and C⋯H/H⋯C contacts make the most significant contributions to the HS, at 26.6%, 20.8%, 15.4% and 8.7%, respectively.

## 5. Database survey

A survey of the Cambridge Structural Database (Version 6.00; April 2025; Groom *et al.*, 2016 ▸) did not reveal any structures of saflufenacil. Three analogous structures containing a tri­fluoro­methyl-substituted 3-methyl-2,6-dioxo-3,6-di­hydro­pyrimidine moiety and a phenyl group were found, namely CSD refcode QANMUO (Li *et al.*, 2005 ▸), RIRZEY (Tian, 2007 ▸), and YOCYEX (Keates, 2019 ▸). The dihedral angles between the pyrimidine ring and the phenyl group in these three structures are 89.14 (10), 68.38 (7), and 85.15 (17)/82.76 (18)°, respectively.

## 6. Synthesis and crystallization

Saflufenacil (raw material, >99% purity) was purchased from Aladdin. It was dissolved in 99.5% aceto­nitrile at 323 K. The hot solution was capped and placed at room temperature. After several hours, single crystals of saflufenacil were obtained.

## 7. Refinement

Crystal data, data collection and structure refinement details are summarized in Table 2 ▸. The N-bound H atom was found in difference-Fourier maps and refined as riding, with *U*_iso_(H) = 1.2*U*_eq_(N). C-bound H atoms were positioned with an idealized geometry and treated using riding models with constrained C—H distances as follows: 0.93 Å for aromatic and 0.96 Å for tertiary H atoms, with *U*_iso_(H) = 1.2*U*_eq_(C).

## Supplementary Material

Crystal structure: contains datablock(s) I. DOI: 10.1107/S2056989026004585/dj2090sup1.cif

Structure factors: contains datablock(s) I. DOI: 10.1107/S2056989026004585/dj2090Isup3.hkl

Supporting information file. DOI: 10.1107/S2056989026004585/dj2090Isup3.cml

CCDC reference: 2551133

Additional supporting information:  crystallographic information; 3D view; checkCIF report

## Figures and Tables

**Table 1:** Hydrogen-bond geometry (Å, °)

*D*—H⋯*A*	*D*—H	H⋯*A*	*D*⋯*A*	*D*—H⋯*A*
N3—H3⋯O2^i^	0.86 (1)	2.06 (1)	2.913 (2)	172 (4)
C14—H14*B*⋯O1^ii^	0.96	2.58	3.492 (3)	159
C17—H17*C*⋯O1^ii^	0.96	2.53	3.475 (4)	170

Symmetry codes: (i) 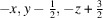; (ii) 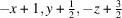.

**Table 2:** Experimental details

Crystal data	
Chemical formula	C_17_H_17_ClF_4_N_4_O_5_S
*M*_r_	500.85
Crystal system, space group	Monoclinic, *P*2_1_/*c*
Temperature (K)	296
*a*, *b*, *c* (Å)	9.4837 (4), 7.7770 (4), 28.4673 (13)
β (°)	95.215 (2)
*V* (Å^3^)	2090.91 (17)
*Z*	4
Radiation type	Ga *K*α, λ = 1.34139 Å
μ (mm^−1^)	2.17
Crystal size (mm)	0.15 × 0.04 × 0.03

Data collection	
Diffractometer	Bruker D8 VENTURE Metaljet
Absorption correction	Multi-scan (*SADABS*; Krause *et al.*, 2015 ▸)
*T*_min_, *T*_max_	0.634, 0.752
No. of measured, independent and observed [*I* > 2σ(*I*)] reflections	16822, 4757, 3870
*R*_int_	0.062
(sin θ/λ)_max_ (Å^−1^)	0.650

Refinement	
*R*[*F*^2^ > 2σ(*F*^2^)], *wR*(*F*^2^), *S*	0.051, 0.135, 1.06
No. of reflections	4757
No. of parameters	296
No. of restraints	1
H-atom treatment	H atoms treated by a mixture of independent and constrained refinement
Δρ_max_, Δρ_min_ (e Å^−3^)	0.30, −0.57

Computer programs: *APEX4* and *SAINT* (Bruker, 2018 ▸), *SHELXT2018/2* (Sheldrick, 2015*a* ▸), *SHELXL2018/3* (Sheldrick, 2015*b* ▸), *ORTEP-3 for Windows* (Farrugia, 2012 ▸) and *OLEX2* (Dolomanov *et al.*, 2009 ▸).

## supplementary crystallographic information

### 2-Chloro-4-fluoro-N-(N-isopropyl-N-methylsulfamoyl)-5-[3-methyl-2,6-dioxo-4-(trifluoromethyl)-1,2,3,6-tetrahydropyrimidin-1-yl]benzamide Crystal data

**Table:**

C_17_H_17_ClF_4_N_4_O_5_S	*F*(000) = 1024
*M**_r_* = 500.85	*D*_x_ = 1.591 Mg m^−^^3^
Monoclinic, *P*2_1_/*c*	Ga *K*α radiation, λ = 1.34139 Å
*a* = 9.4837 (4) Å	Cell parameters from 5773 reflections
*b* = 7.7770 (4) Å	θ = 4.7–60.4°
*c* = 28.4673 (13) Å	µ = 2.17 mm^−^^1^
β = 95.215 (2)°	*T* = 296 K
*V* = 2090.91 (17) Å^3^	Block, colourless
*Z* = 4	0.15 × 0.04 × 0.03 mm

### 2-Chloro-4-fluoro-N-(N-isopropyl-N-methylsulfamoyl)-5-[3-methyl-2,6-dioxo-4-(trifluoromethyl)-1,2,3,6-tetrahydropyrimidin-1-yl]benzamide Data collection

**Table:**

Bruker D8 VENTURE Metaljet diffractometer	4757 independent reflections
Radiation source: liquid Metal X-ray source, Bruker Excillum Metaljet D2+	3870 reflections with *I* > 2σ(*I*)
Mirror optics monochromator	*R*_int_ = 0.062
Detector resolution: 7.41 pixels mm^-1^	θ_max_ = 60.7°, θ_min_ = 2.7°
ω and φ scans	*h* = −12→11
Absorption correction: multi-scan (SADABS; Krause *et al.*, 2015)	*k* = −10→8
*T*_min_ = 0.634, *T*_max_ = 0.752	*l* = −37→35
16822 measured reflections	

### 2-Chloro-4-fluoro-N-(N-isopropyl-N-methylsulfamoyl)-5-[3-methyl-2,6-dioxo-4-(trifluoromethyl)-1,2,3,6-tetrahydropyrimidin-1-yl]benzamide Refinement

**Table:**

Refinement on *F*^2^	Primary atom site location: structure-invariant direct methods
Least-squares matrix: full	Hydrogen site location: mixed
*R*[*F*^2^ > 2σ(*F*^2^)] = 0.051	H atoms treated by a mixture of independent and constrained refinement
*wR*(*F*^2^) = 0.135	*w* = 1/[σ^2^(*F*_o_^2^) + (0.0627*P*)^2^ + 0.6864*P*] where *P* = (*F*_o_^2^ + 2*F*_c_^2^)/3
*S* = 1.06	(Δ/σ)_max_ = 0.001
4757 reflections	Δρ_max_ = 0.30 e Å^−^^3^
296 parameters	Δρ_min_ = −0.57 e Å^−^^3^
1 restraint	

### 2-Chloro-4-fluoro-N-(N-isopropyl-N-methylsulfamoyl)-5-[3-methyl-2,6-dioxo-4-(trifluoromethyl)-1,2,3,6-tetrahydropyrimidin-1-yl]benzamide Special details

**Table:**

Geometry. All esds (except the esd in the dihedral angle between two l.s. planes) are estimated using the full covariance matrix. The cell esds are taken into account individually in the estimation of esds in distances, angles and torsion angles; correlations between esds in cell parameters are only used when they are defined by crystal symmetry. An approximate (isotropic) treatment of cell esds is used for estimating esds involving l.s. planes.

### 2-Chloro-4-fluoro-N-(N-isopropyl-N-methylsulfamoyl)-5-[3-methyl-2,6-dioxo-4-(trifluoromethyl)-1,2,3,6-tetrahydropyrimidin-1-yl]benzamide Fractional atomic coordinates and isotropic or equivalent isotropic displacement parameters (Å^2^)

**Table:**

	*x*	*y*	*z*	*U*_iso_*/*U*_eq_	
Cl1	0.15726 (7)	−0.22265 (7)	0.75754 (2)	0.05320 (17)	
S1	0.41955 (5)	0.08312 (6)	0.86482 (2)	0.04185 (15)	
F1	0.2631 (3)	0.8098 (3)	0.51863 (7)	0.1289 (10)	
F2	0.1016 (2)	0.6809 (3)	0.47590 (5)	0.0938 (6)	
F3	0.0508 (4)	0.8668 (3)	0.52622 (9)	0.1486 (12)	
F4	−0.04522 (14)	0.08440 (18)	0.61300 (5)	0.0580 (4)	
O1	0.26931 (19)	0.1929 (2)	0.58162 (6)	0.0586 (4)	
O2	−0.01763 (16)	0.5228 (2)	0.66334 (6)	0.0524 (4)	
O3	0.46854 (15)	0.1578 (3)	0.76522 (5)	0.0646 (5)	
O4	0.53719 (19)	−0.0253 (2)	0.85884 (6)	0.0627 (4)	
O5	0.32650 (19)	0.0453 (2)	0.89983 (5)	0.0606 (4)	
N1	0.21468 (19)	0.4603 (3)	0.55298 (6)	0.0480 (4)	
N2	0.13257 (15)	0.3613 (2)	0.62373 (5)	0.0339 (3)	
N3	0.30911 (17)	0.0776 (2)	0.81556 (5)	0.0434 (4)	
N4	0.47605 (17)	0.2753 (2)	0.87183 (6)	0.0424 (4)	
C1	0.2099 (2)	0.3278 (3)	0.58558 (6)	0.0402 (4)	
C2	0.1328 (2)	0.6045 (3)	0.55645 (7)	0.0473 (5)	
C3	0.0512 (2)	0.6287 (3)	0.59116 (7)	0.0473 (5)	
H3A	−0.005284	0.726504	0.591119	0.057*	
C4	0.04813 (19)	0.5062 (2)	0.62899 (7)	0.0372 (4)	
C5	0.3111 (4)	0.4303 (6)	0.51634 (11)	0.0935 (12)	
H5A	0.268592	0.350698	0.493493	0.140*	
H5B	0.398620	0.383601	0.530459	0.140*	
H5C	0.329277	0.537136	0.501082	0.140*	
C6	0.1380 (4)	0.7402 (4)	0.51875 (10)	0.0749 (8)	
C7	0.13563 (18)	0.2288 (2)	0.65892 (6)	0.0335 (4)	
C8	0.0455 (2)	0.0896 (2)	0.65223 (6)	0.0381 (4)	
C9	0.0479 (2)	−0.0459 (2)	0.68336 (7)	0.0398 (4)	
H9	−0.014175	−0.137955	0.678205	0.048*	
C10	0.1453 (2)	−0.0409 (2)	0.72254 (6)	0.0355 (4)	
C11	0.23664 (19)	0.0982 (2)	0.73126 (6)	0.0346 (4)	
C12	0.23085 (19)	0.2322 (2)	0.69850 (6)	0.0357 (4)	
H12	0.292139	0.325114	0.703451	0.043*	
C13	0.3499 (2)	0.1113 (3)	0.77162 (6)	0.0405 (4)	
C14	0.6130 (2)	0.3254 (4)	0.85613 (10)	0.0576 (6)	
H14A	0.670870	0.225101	0.853782	0.086*	
H14B	0.659388	0.404079	0.878481	0.086*	
H14C	0.598454	0.380058	0.825834	0.086*	
C15	0.3725 (2)	0.4126 (3)	0.88052 (8)	0.0497 (5)	
H15	0.279005	0.358768	0.880373	0.060*	
C16	0.3641 (4)	0.5460 (4)	0.84222 (13)	0.0886 (10)	
H16A	0.454678	0.600633	0.841479	0.133*	
H16B	0.294374	0.630583	0.848338	0.133*	
H16C	0.338001	0.492082	0.812357	0.133*	
C17	0.4089 (4)	0.4893 (5)	0.92897 (12)	0.0826 (9)	
H17A	0.408720	0.400371	0.952359	0.124*	
H17B	0.340020	0.575101	0.934964	0.124*	
H17C	0.501087	0.541044	0.930323	0.124*	
H3	0.2223 (16)	0.053 (5)	0.8196 (14)	0.099*	

### 2-Chloro-4-fluoro-N-(N-isopropyl-N-methylsulfamoyl)-5-[3-methyl-2,6-dioxo-4-(trifluoromethyl)-1,2,3,6-tetrahydropyrimidin-1-yl]benzamide Atomic displacement parameters (Å^2^)

**Table:**

	*U*^11^	*U*^22^	*U*^33^	*U*^12^	*U*^13^	*U*^23^
Cl1	0.0822 (4)	0.0361 (3)	0.0416 (3)	−0.0026 (2)	0.0072 (2)	0.01049 (19)
S1	0.0518 (3)	0.0430 (3)	0.0298 (2)	−0.0025 (2)	−0.00164 (18)	0.00665 (17)
F1	0.184 (2)	0.1263 (19)	0.0737 (12)	−0.0961 (19)	−0.0038 (13)	0.0371 (12)
F2	0.1367 (16)	0.0974 (13)	0.0422 (8)	−0.0187 (12)	−0.0198 (9)	0.0245 (8)
F3	0.267 (4)	0.0759 (14)	0.1087 (17)	0.0604 (18)	0.049 (2)	0.0549 (13)
F4	0.0584 (7)	0.0652 (8)	0.0466 (7)	−0.0156 (6)	−0.0160 (6)	0.0094 (6)
O1	0.0729 (10)	0.0564 (10)	0.0492 (9)	0.0207 (8)	0.0198 (8)	0.0006 (7)
O2	0.0537 (8)	0.0534 (9)	0.0526 (9)	0.0111 (7)	0.0185 (7)	0.0011 (7)
O3	0.0402 (8)	0.1143 (15)	0.0396 (8)	−0.0170 (9)	0.0060 (6)	0.0086 (9)
O4	0.0703 (10)	0.0530 (9)	0.0616 (10)	0.0192 (8)	−0.0111 (8)	0.0020 (8)
O5	0.0817 (11)	0.0686 (11)	0.0319 (7)	−0.0242 (9)	0.0068 (7)	0.0098 (7)
N1	0.0510 (9)	0.0591 (11)	0.0349 (8)	−0.0002 (8)	0.0099 (7)	0.0109 (8)
N2	0.0377 (7)	0.0333 (7)	0.0313 (7)	0.0035 (6)	0.0057 (6)	0.0055 (6)
N3	0.0412 (8)	0.0604 (11)	0.0286 (7)	−0.0085 (8)	0.0030 (6)	0.0049 (7)
N4	0.0380 (8)	0.0431 (9)	0.0462 (9)	−0.0026 (7)	0.0049 (7)	−0.0008 (7)
C1	0.0436 (9)	0.0473 (11)	0.0304 (8)	0.0031 (8)	0.0065 (7)	0.0039 (8)
C2	0.0605 (12)	0.0409 (10)	0.0386 (10)	−0.0075 (9)	−0.0053 (9)	0.0094 (8)
C3	0.0624 (12)	0.0349 (10)	0.0434 (10)	0.0061 (9)	−0.0015 (9)	0.0073 (8)
C4	0.0389 (9)	0.0334 (9)	0.0392 (9)	0.0007 (7)	0.0037 (7)	0.0011 (7)
C5	0.099 (2)	0.129 (3)	0.0592 (17)	0.023 (2)	0.0472 (17)	0.0280 (18)
C6	0.119 (2)	0.0560 (15)	0.0480 (13)	−0.0129 (16)	0.0003 (14)	0.0202 (11)
C7	0.0381 (9)	0.0318 (8)	0.0310 (8)	0.0014 (7)	0.0059 (7)	0.0046 (6)
C8	0.0397 (9)	0.0400 (10)	0.0338 (9)	−0.0025 (8)	−0.0003 (7)	0.0010 (7)
C9	0.0470 (10)	0.0343 (9)	0.0385 (9)	−0.0080 (8)	0.0059 (8)	−0.0002 (7)
C10	0.0454 (9)	0.0313 (9)	0.0313 (8)	0.0026 (7)	0.0111 (7)	0.0041 (7)
C11	0.0378 (8)	0.0390 (9)	0.0278 (8)	−0.0007 (7)	0.0063 (6)	0.0013 (7)
C12	0.0399 (9)	0.0357 (9)	0.0321 (8)	−0.0035 (7)	0.0058 (7)	0.0006 (7)
C13	0.0402 (9)	0.0509 (11)	0.0308 (8)	−0.0033 (8)	0.0056 (7)	0.0054 (8)
C14	0.0381 (10)	0.0663 (15)	0.0690 (15)	−0.0073 (10)	0.0079 (10)	−0.0055 (12)
C15	0.0440 (10)	0.0483 (12)	0.0582 (13)	0.0021 (9)	0.0128 (9)	−0.0020 (10)
C16	0.104 (2)	0.0696 (19)	0.096 (2)	0.0323 (18)	0.028 (2)	0.0273 (17)
C17	0.0833 (19)	0.093 (2)	0.0739 (19)	0.0020 (17)	0.0210 (15)	−0.0303 (17)

### 2-Chloro-4-fluoro-N-(N-isopropyl-N-methylsulfamoyl)-5-[3-methyl-2,6-dioxo-4-(trifluoromethyl)-1,2,3,6-tetrahydropyrimidin-1-yl]benzamide Geometric parameters (Å, º)

**Table:**

Cl1—C10	1.7269 (18)	C3—C4	1.440 (3)
S1—O4	1.4210 (17)	C5—H5A	0.9600
S1—O5	1.4207 (16)	C5—H5B	0.9600
S1—N3	1.6727 (16)	C5—H5C	0.9600
S1—N4	1.5940 (18)	C7—C8	1.382 (3)
F1—C6	1.305 (4)	C7—C12	1.379 (3)
F2—C6	1.320 (3)	C8—C9	1.376 (3)
F3—C6	1.315 (4)	C9—H9	0.9300
F4—C8	1.347 (2)	C9—C10	1.383 (3)
O1—C1	1.201 (3)	C10—C11	1.394 (3)
O2—C4	1.214 (2)	C11—C12	1.396 (3)
O3—C13	1.211 (2)	C11—C13	1.503 (3)
N1—C1	1.390 (3)	C12—H12	0.9300
N1—C2	1.373 (3)	C14—H14A	0.9600
N1—C5	1.467 (3)	C14—H14B	0.9600
N2—C1	1.389 (2)	C14—H14C	0.9600
N2—C4	1.398 (2)	C15—H15	0.9800
N2—C7	1.435 (2)	C15—C16	1.502 (4)
N3—C13	1.368 (2)	C15—C17	1.514 (4)
N3—H3	0.862 (10)	C16—H16A	0.9600
N4—C14	1.464 (3)	C16—H16B	0.9600
N4—C15	1.487 (3)	C16—H16C	0.9600
C2—C3	1.323 (3)	C17—H17A	0.9600
C2—C6	1.509 (3)	C17—H17B	0.9600
C3—H3A	0.9300	C17—H17C	0.9600
			
O4—S1—N3	108.84 (10)	C12—C7—C8	118.58 (16)
O4—S1—N4	108.23 (10)	F4—C8—C7	118.57 (16)
O5—S1—O4	120.38 (12)	F4—C8—C9	118.85 (17)
O5—S1—N3	101.61 (9)	C9—C8—C7	122.55 (17)
O5—S1—N4	109.22 (10)	C8—C9—H9	121.0
N4—S1—N3	107.88 (9)	C8—C9—C10	117.95 (17)
C1—N1—C5	114.7 (2)	C10—C9—H9	121.0
C2—N1—C1	120.31 (17)	C9—C10—Cl1	116.52 (14)
C2—N1—C5	125.0 (2)	C9—C10—C11	121.59 (16)
C1—N2—C4	125.67 (15)	C11—C10—Cl1	121.70 (14)
C1—N2—C7	115.61 (15)	C10—C11—C12	118.32 (16)
C4—N2—C7	118.63 (14)	C10—C11—C13	125.29 (17)
S1—N3—H3	115 (3)	C12—C11—C13	116.23 (16)
C13—N3—S1	123.65 (14)	C7—C12—C11	120.98 (17)
C13—N3—H3	121 (3)	C7—C12—H12	119.5
C14—N4—S1	120.59 (16)	C11—C12—H12	119.5
C14—N4—C15	118.50 (19)	O3—C13—N3	122.58 (18)
C15—N4—S1	118.33 (14)	O3—C13—C11	120.94 (17)
O1—C1—N1	122.74 (18)	N3—C13—C11	116.38 (16)
O1—C1—N2	121.51 (18)	N4—C14—H14A	109.5
N2—C1—N1	115.75 (17)	N4—C14—H14B	109.5
N1—C2—C6	117.7 (2)	N4—C14—H14C	109.5
C3—C2—N1	122.89 (18)	H14A—C14—H14B	109.5
C3—C2—C6	119.4 (2)	H14A—C14—H14C	109.5
C2—C3—H3A	119.4	H14B—C14—H14C	109.5
C2—C3—C4	121.14 (19)	N4—C15—H15	107.7
C4—C3—H3A	119.4	N4—C15—C16	111.6 (2)
O2—C4—N2	120.91 (17)	N4—C15—C17	109.6 (2)
O2—C4—C3	125.35 (19)	C16—C15—H15	107.7
N2—C4—C3	113.73 (17)	C16—C15—C17	112.4 (3)
N1—C5—H5A	109.5	C17—C15—H15	107.7
N1—C5—H5B	109.5	C15—C16—H16A	109.5
N1—C5—H5C	109.5	C15—C16—H16B	109.5
H5A—C5—H5B	109.5	C15—C16—H16C	109.5
H5A—C5—H5C	109.5	H16A—C16—H16B	109.5
H5B—C5—H5C	109.5	H16A—C16—H16C	109.5
F1—C6—F2	107.7 (3)	H16B—C16—H16C	109.5
F1—C6—F3	105.9 (3)	C15—C17—H17A	109.5
F1—C6—C2	112.3 (3)	C15—C17—H17B	109.5
F2—C6—C2	113.0 (2)	C15—C17—H17C	109.5
F3—C6—F2	107.2 (3)	H17A—C17—H17B	109.5
F3—C6—C2	110.3 (3)	H17A—C17—H17C	109.5
C8—C7—N2	119.56 (16)	H17B—C17—H17C	109.5
C12—C7—N2	121.79 (16)		
			
Cl1—C10—C11—C12	173.02 (14)	C3—C2—C6—F1	115.8 (3)
Cl1—C10—C11—C13	−2.2 (3)	C3—C2—C6—F2	−122.1 (3)
S1—N3—C13—O3	−4.3 (3)	C3—C2—C6—F3	−2.1 (4)
S1—N3—C13—C11	179.30 (14)	C4—N2—C1—O1	−172.92 (19)
S1—N4—C15—C16	−118.2 (2)	C4—N2—C1—N1	7.6 (3)
S1—N4—C15—C17	116.6 (2)	C4—N2—C7—C8	94.3 (2)
F4—C8—C9—C10	177.70 (17)	C4—N2—C7—C12	−88.8 (2)
O4—S1—N3—C13	−53.8 (2)	C5—N1—C1—O1	−6.8 (3)
O4—S1—N4—C14	20.6 (2)	C5—N1—C1—N2	172.7 (2)
O4—S1—N4—C15	−177.87 (15)	C5—N1—C2—C3	−177.5 (3)
O5—S1—N3—C13	178.16 (19)	C5—N1—C2—C6	2.4 (4)
O5—S1—N4—C14	153.35 (17)	C6—C2—C3—C4	−177.1 (2)
O5—S1—N4—C15	−45.13 (18)	C7—N2—C1—O1	3.4 (3)
N1—C2—C3—C4	2.7 (3)	C7—N2—C1—N1	−176.01 (16)
N1—C2—C6—F1	−64.0 (3)	C7—N2—C4—O2	2.5 (3)
N1—C2—C6—F2	58.0 (4)	C7—N2—C4—C3	−178.81 (17)
N1—C2—C6—F3	178.0 (3)	C7—C8—C9—C10	−0.3 (3)
N2—C7—C8—F4	−1.4 (3)	C8—C7—C12—C11	0.0 (3)
N2—C7—C8—C9	176.67 (17)	C8—C9—C10—Cl1	−173.66 (15)
N2—C7—C12—C11	−177.02 (16)	C8—C9—C10—C11	1.5 (3)
N3—S1—N4—C14	−97.00 (18)	C9—C10—C11—C12	−1.9 (3)
N3—S1—N4—C15	64.52 (17)	C9—C10—C11—C13	−177.16 (17)
N4—S1—N3—C13	63.4 (2)	C10—C11—C12—C7	1.1 (3)
C1—N1—C2—C3	2.8 (3)	C10—C11—C13—O3	133.1 (2)
C1—N1—C2—C6	−177.4 (2)	C10—C11—C13—N3	−50.5 (3)
C1—N2—C4—O2	178.75 (19)	C12—C7—C8—F4	−178.46 (17)
C1—N2—C4—C3	−2.5 (3)	C12—C7—C8—C9	−0.4 (3)
C1—N2—C7—C8	−82.4 (2)	C12—C11—C13—O3	−42.2 (3)
C1—N2—C7—C12	94.6 (2)	C12—C11—C13—N3	134.22 (19)
C2—N1—C1—O1	173.0 (2)	C13—C11—C12—C7	176.79 (17)
C2—N1—C1—N2	−7.5 (3)	C14—N4—C15—C16	43.7 (3)
C2—C3—C4—O2	175.8 (2)	C14—N4—C15—C17	−81.5 (3)
C2—C3—C4—N2	−2.8 (3)		

### 2-Chloro-4-fluoro-N-(N-isopropyl-N-methylsulfamoyl)-5-[3-methyl-2,6-dioxo-4-(trifluoromethyl)-1,2,3,6-tetrahydropyrimidin-1-yl]benzamide Hydrogen-bond geometry (Å, º)

**Table:**

*D*—H···*A*	*D*—H	H···*A*	*D*···*A*	*D*—H···*A*
C14—H14*B*···O1^i^	0.96	2.58	3.492 (3)	159
C17—H17*C*···O1^i^	0.96	2.53	3.475 (4)	170
N3—H3···O2^ii^	0.86 (1)	2.06 (1)	2.913 (2)	172 (4)

Symmetry codes: (i) −*x*+1, *y*+1/2, −*z*+3/2; (ii) −*x*, *y*−1/2, −*z*+3/2.
